# Rate and correlates of post-traumatic stress disorder (PTSD) following the Beirut blast and the economic crisis among Lebanese University students: a cross-sectional study

**DOI:** 10.1186/s12888-022-04180-y

**Published:** 2022-08-05

**Authors:** Christian-Joseph El Zouki, Abdallah Chahine, Mariam Mhanna, Sahar Obeid, Souheil Hallit

**Affiliations:** 1 School of Medicine and Medical Sciences, Holy Spirit University of Kaslik, P.O. Box 446, Jounieh, Lebanon; 2 School of Arts and Sciences, Social and Education Sciences Department, Lebanese American University, Jbeil, Lebanon; 3 Psychology Department, College of Humanities, Effat University, Jeddah, 21478 Saudi Arabia; 4 Research Department, Psychiatric Hospital of the Cross, Jal Eddib, Lebanon

**Keywords:** Post-traumatic stress disorder, Correlates, University students, Lebanon

## Abstract

**Background:**

Post-traumatic stress disorder (PTSD) is a mental illness that develops in some people after they have experienced a stunning, scary, or dangerous incident. Due to major disasters like as the Economic Crisis and the Beirut Blast, Lebanese people are struggling with a variety of mental health issues. The study objectives were to find the rate of PTSD and its association with stress, anxiety, depression, financial well-being and coping strategies among university students in Lebanon.

**Methods:**

This is a cross-sectional study, conducted between May and August 2021, which enrolled 419 university students from all districts of Lebanon. The PTSD Checklist-Specific Version (PCL-S) was used to evaluate manifestation of PSTD.

**Results:**

The results showed that 132 (31.5%), 109 (26.0%) and 169 (40.3%) had PTSD from COVID, Beirut blast and economic crisis respectively. More avoidant coping (Beta = 0.52) and more anxiety (Beta = 0.62) were significantly associated with more PTSD from the Beirut Blast. More avoidant coping (Beta = 0.56), depression (Beta = 0.40) and anxiety (Beta = 0.49) were significantly associated with more PTSD from the economic crisis, whereas more financial wellbeing (Beta = − 0.31) was significantly associated with less PTSD from the economic crisis.

**Conclusion:**

Significant rates of PTSD were found in our sample of Lebanese university students, whether from the Beirut blast, or from the current economic crisis. Significant correlations of these PTSD rates were found with factors such as avoidant coping, depression, anxiety and financial wellbeing. Such findings must raise the attention to serious mental and psychosocial alteration endured by Lebanese youth that are still under fatal cumulative traumatic events, that were and even may be, intergenerationally and unintentionally transmissible, therefore, affecting not only the present, but also the future of a whole nation.

## Background

Post-traumatic stress disorder (PTSD) is a mental illness that develops in some people after they have experienced a stunning, scary, or dangerous incident. It establishes itself in both psychological and physical manifestations. The Diagnostic and Statistical Manual of Mental Disorders (DSM-5) defines PTSD as a trauma and stressor-related disorder with symptoms that last more than one month [1]. Psychological symptoms like nightmares [2] and intrusive thoughts are common in PTSD patients, but they can also exhibit symptoms such as hypervigilance, and being constantly on the lookout [3]. Hyperarousal can also be present, which can be sparked by any trigger [4]. Patients prefer to avoid places, people, and situations that bring up memories of the underlying trauma in order to stay away from reliving the memories of the experience [1]. Other functional and social consequences of this persistent attempt to avoid stressors and constant replay of events include sleep disturbances [5] and general irritability [6].

It is important to highlight that not all stressful situations result in the onset of PTSD [7]. A number of factors play a crucial role in determining whether or not individuals may develop PTSD, but one distinguishing aspect is the type of trauma they have experienced [8]. In contrast to occurrences like accidents or disasters, if the trauma has a more personal aspect, such as rape, the patient is more prone to acquire PTSD [9]. Another component is the coping tactics used, which can reduce future PTSD symptoms by, for example, offering emotional support to the patient [10]. Biological causes, such as a malfunction in the endogenous opioid system [11] or a deficit in the arousal and sleep regulating systems, can also cause PTSD [1]. Moreover, genetic predisposition to mood and anxiety disorders, which can be detected in a patient’s family history, can be a contributor in PTSD [12]. Anxiety and depression were found to be comorbid in many investigations of PTSD patients. Depression, anxiety, and phobic avoidance severity levels were all higher among patients suffering from Post-Traumatic Stress [13, 14].

A recent study conducted in Bangladesh showed that people living in poor conditions with low income had higher rates of PTSD. This correlation shows that financial wellbeing might play a major mediating role in people suffering of the repercussions of trauma [15].

Coping with trauma can be challenging, and how well a person can deal with trauma is a key factor in determining the outcome of their rehabilitation after a traumatic experience. People adopt many coping styles during psychological stress [16, 17].

Many coping styles have been previously analyzed, some of them include avoidant coping and approach coping. When dealing with unpleasant conditions, avoidance coping involves cognitive and behavioral strategies aimed at denying, dismissing, or in some other way avoiding stressors. Approach coping is characterized as actively addressing a stressor and look for info, social support, and make an effort to fix certain difficulties [18–20].

Avoidant coping methods were found to be linked to the establishment of PTSD symptoms [21].

Facing internal and external changes, the human body maintains homeostasis by a normal mechanism, called the stress response [22]. The duration and the source of exposure play a major role in late adaptive responses to stress. To a controllable stress trigger, a delicate behavioral approach is obtained that prepares well the organism for the subsequent encounter. However, if the source cannot be controlled, a more complex reorganization of neurons is needed in the brain, leading to the development of new coping techniques to deal with the stress source [23].

Lebanon, a Middle Eastern country, is in the midst of constant regional turmoil and adjacent warzones. Lebanese people are plagued with both macro and micro-traumas which puts them at higher risk of having PTSD [24]. The prevalence of PTSD in teenagers who went through the Lebanese 2006 July War, ranged from 15.4 to 35.0% [25].

Subjects in late adolescence-early adulthood (16- to 25-year-olds) seem to be at a higher risk of experiencing potentially traumatic events and/or developing PTSD symptoms [26]. In a study targeting a large sample of newly matriculated US college students [27], about 9% of the 3014 students have met criteria for PTSD. As explored by a recent study among University students in China, 14.1% of college students who experienced a major earthquake, developed PTSD [28]. Lebanese university students have survived constant Middle Eastern changes [24], and a global COVID-19 pandemic [29], adding to the potentially stressful time experienced by undergraduate students while transitioning to university [30], that’s why opting for avoidance as a potential coping mechanism would seem possible.

On August 4th 2020, one of the most powerful blasts ever recorded in contemporary history shook Beirut, the capital of Lebanon [31], which further strained the socio-economic situation in the country [32], and created severe psychological distress within Lebanese citizens [33]. Due to major disasters like as the Economic Crisis and the Beirut Blast, Lebanese people are struggling with a variety of mental health issues [29].

Adding to the above mentioned potential traumatic experiences, it has been demonstrated that mental illness is becoming very common among university students [34, 35]. In addition to academic stress, students are taking on many obligations and transitioning to adulthood with all its responsibilities and challenges, without being equipped with the necessary skills and maturity of adulthood [36]. Consequently, studying PTSD and its correlates in a sample of Lebanese university students would seem crucial.

Our study was guided by those three research questions: (1) What is the prevalence of PTSD among university students in Lebanon? (2) Which coping strategies are of highest relevance when it comes to Post Traumatic Symptoms? and (3) Is there a significant correlation between PTSD and financial well-being, stress, anxiety, and depression?

We evaluated the intensity of Post Traumatic Symptoms and its correlates based on a survey of college students. We initially hypothesized that PTSD levels will be elevated in our sample, and that it will be correlated with higher levels of stress, depression, anxiety and poor financial well-being, as well as negative coping strategies such as avoidant coping. To the best of our knowledge, little is known about the correlation between stress, anxiety, depression, financial well-being, coping strategies and PTSD among university students in Lebanon. Thus, the aim of this cross-sectional study was to find the rate and correlates of PTSD in Lebanese university students amid the socioeconomic crisis and in the aftermath of the Beirut port explosion.

## Methods

### Study design

Between May and August 2021, 419 university students from various Lebanese universities participated in this cross-sectional research. No inclusion or exclusion criteria were defined during the recruitment procedure, but we demanded that all participants should be university students that were in Lebanon during the Beirut Blast and the Economic Crisis. The questionnaire was developed with Google Forms and distributed to participants via social media platforms and messaging applications. Data collection was done using snowball sampling and respondent-driven procedures due to the severe coronavirus pandemic imposing social distancing and the closure of all universities in Lebanon. Individual subjects received study objectives and general instructions online prior to participation. No credits were received for participation.

### Minimal sample size calculation

The G-power software calculated a minimal sample of 395 students, based on an effect size f^2^ = 2%, α error = 5%, power = 80%, and a maximum of 15 factors to be included in the multivariable analysis.

### Questionnaire

The survey was developed in English, which is widely used in Lebanon and specially among university students. Furthermore, we used English because it’s the most adopted language by Lebanese Universities at academic and teaching levels. The questionnaire was divided in three sections. The first part contained a written consent form that confirmed the participants’ permission to voluntarily complete the survey, in addition to other ethical considerations such as the insurance of the confidentiality and anonymity of the respondents. Moreover, the first part also included general information about the study and some essential instructions for the questionnaire. The second segment includes questions that evaluate the socio-demographic data of the participants (age, gender, district of residence, etc.). The survey also included questions about the presence of a personal history of PTSD, Depression and Anxiety Disorders. The Household Crowding Index (HCI) was calculated by dividing the number of people living in the house by the number of rooms in the house, which represents the family’s socioeconomic status (SES). A greater HCI indicates a lower SES. The third section of the survey comprised the following measures:

### PTSD checklist- specific version (PCL-S)

This questionnaire was employed to evaluate the manifestations of PTSD according to the DSM-4 [37]. It has 17 items that are scored from 1 (not at all bothersome) to 5 (extremely bothersome); higher results imply greater severity. The PCL-S is a variation of the PCL-C (Civilian) that focuses on a specific “traumatic experience”, as opposed to stressful experiences in general, as in the PCL-C. In this study the PCL-S was used for the Lebanese economic crisis and the Beirut Blast respectively (current Cronbach’s α = 0.945 for COVID-19 pandemic, 0.947 for the Beirut blast and 0.959 for the economic crisis). Scores ≥44 indicate the presence of PTSD [38].

### Financial wellbeing scale

This scale indicates how secure and safe a person feels regarding his financial situation. It is composed of 8 items rated from 1 to 10, with higher scores indicating the least pressure felt from the current financial situation in the country [39]. We obtained a written permission to use this scale (current Cronbach’s α = 0.931).

### Beirut distress scale (BDS-10)

This short tool is valid and reliable to screen and recognize psychological distress in the Lebanese population [40]. It is made up of 10 items rated from 0 (never) to 3 (very much); higher scores indicate higher distress (current Cronbach’s α = 0.886).

### Lebanese anxiety scale (LAS)

This is a 10-item scale used to rule out anxiety. 7 questions are rated from 0 to 4 and 3 questions graded from 1 to 4; higher scores indicate more anxiety [41, 42] (current Cronbach’s α = 0.907).

### Patient health questionnaire (PHQ-9)

It is a self-administered multipurpose scale that assesses, screens and measures the severity of depression during the previous two weeks. This instrument is validated in Lebanon [43], and it’s made of 9 questions rated from “0” (not at all) to “3” (nearly every day); higher scores indicate higher depressive symptoms (current Cronbach’s α = 0.887).

### Brief coping orientation to problems experiences (brief-COPE)

It is an abbreviated, 28-item self-report scale deriving from the COPE (Coping Orientation to Problems Experienced) Inventory [44]. Scores are presented under two subscales (Avoidant Coping and Approach Coping). The questions are rated from “1” (I haven’t been doing this at all) to “4” (I’ve been doing this a lot). The scale can determine someone’s primary coping styles as either Approach Coping, or Avoidant Coping [44], with higher scores indicating more coping strategies (current Cronbach’s α = 0.856 for approach coping and 0.790 for avoidant coping).

### Statistical analysis

Statistical analysis was performed using SPSS software, version 23. There was no missing data since all questions were required in the Google form. Both PTSD scores had a normal distribution (skewness and kurtosis between − 1 and + 1 [45]). Accordingly, the Student t-test was used to check for an association between the PTSD scores and dichotomous variables (i.e., gender), while the Pearson correlation test was used to correlate two continuous variables (i.e., age, household crowding index, etc.). Effect sizes were calculated for all bivariate analyses; in psychological research, values of 0.1 were deemed to have small effect size, whereas values of 0.2 and 0.3 were classified as having medium and large effect sizes respectively [46]. Stepwise linear regressions were conducted taking the PTSD scores as dependent variables. Covariates that were included in the final models were those that showed an effect size or correlation > │0.24│ in the bivariate analysis to have parsimonious models [47]. *P* < 0.05 was considered significant.

## Results

The sample consisted of 419 participants, with a mean age of 21.02 ± 2.59 years and 70.4% females. Other characteristics and description of the scores can be found in Table 1.

**Table 1:** Sociodemographic characteristics of the participants (*N* = 419)

Variable	N (%)
Gender	
Male	124 (29.6)
Female	295 (70.4%)
Personal history of anxiety disorders (yes)	83 (19.8%)
Personal history of PTSD (yes)	26 (6.2%)
Personal history of depression (yes)	82 (19.6%)
	**Mean ± SD**
Age (in years)	21.02 ± 2.59
Household crowding index	0.91 ± 0.42
Financial wellbeing scale	39.19 ± 16.61
Depression (PHQ9 score)	11.24 ± 6.60
Anxiety (LAS score)	17.11 ± 8.81
Stress (BDS score)	13.79 ± 7.04
PTSD from Beirut Blast	36.25 ± 15.73
PTSD from economic crisis	42.22 ± 17.97

Based on the cutoff value of 44 for the PCL-S scale, the results showed that 132 (31.5%), 109 (26.0%) and 169 (40.3%) had PTSD from COVID, Beirut blast and economic crisis respectively.

### Bivariate analysis

Higher depression, anxiety, stress, avoidant coping and coping approach were significantly associated with more PTSD from the Beirut blast and from the economic crisis, whereas higher financial wellbeing was significantly associated with less PTSD from the Beirut blast and from the economic crisis. Furthermore, higher household crowding index was significantly associated with more PTSD from the economic crisis only (Table 2). On another hand, higher mean PTSD from the Beirut blast and from the economic crisis scores were found in those who have a personal history of anxiety, PTSD and depression compared to those who do not. Finally, higher PTSD form the Beirut blast was seen in females compared to males (Table 3).

**Table 2:** Correlation between PTSD scores and other continuous variables

Variable	PTSD from Beirut Blast	PTSD from economic crisis
Age	r = 0.05; *p* = 0.330 [− 0.05; 0.14]	r = 0.01; *p* = 0.785 [− 0.08; 0.11]
Household crowding index	r = 0.09; *p* = 0.075 [− 0.01; 0.18]	r = 0.12; **r = 0.016** [0.02; 0.21]
Financial wellbeing scale	r = −0.31; ***p*** **< 0.001** [− 0.40; − 0.22]	r = − 0.57; ***p*** **< 0.001** [− 0.65; − 0.49]
Depression (PHQ-9 score)	r = 0.54; ***p*** **< 0.001** [0.46; 0.62]	r = 0.68; ***p*** **< 0.001** [0.61; 0.75]
Anxiety (LAS score)	r = 0.61; ***p*** **< 0.001** [0.53; 0.69]	r = 0.71; ***p*** **< 0.001** [0.64; 0.78]
Stress (BDS score)	r = 0.56; ***p*** **< 0.001** [0.48; 0.64]	r = 0.66; ***p*** **< 0.001** [0.59; 0.74]
Avoidant coping	r = 0.54; ***p*** **< 0.001** [0.46; 0.62]	r = 0.61; ***p*** **< 0.001** [0.54; 0.69]
Approach coping	r = 0.15; ***p*** **= 0.002** [0.06; 0.25]	r = 0.13; ***p*** **< 0.001** [0.04; 0.23]

Numbers in bold indicate significant *p*-values. r = Pearson correlation coefficient; p = *p*-value; numbers between brackets refer to the 95% confidence interval

**Table 3:** Correlation between the PTSD scores and other categorical variables

	PTSD from Beirut Blast			PTSD from economic crisis		
Variable	Mean ± SD	***p***	Effect size	Mean ± SD	***p***	Effect size
**Gender**		**0.039**	0.224		0.083	0.184
Male	33.81 ± 15.01			39.87 ± 18.49		
Female	37.28 ± 15.93			43.21 ± 17.68		
**Personal history of anxiety disorders**		**< 0.001**	0.515		**< 0.001**	0.703
No	34.59 ± 14.78			39.72 ± 16.80		
Yes	42.98 ± 17.65			52.36 ± 19.05		
**Personal history of post-traumatic stress disorder**			**0.019**	0.562	**0.002**	0.609
No	35.63 ± 15.22			41.51 ± 17.65		
Yes	45.65 ± 20.09			52.92 ± 19.74		
**Personal history of depression**		**< 0.001**	0.619		**< 0.001**	0.654
No	34.30 ± 14.68			39.89 ± 16.81		
Yes	44.26 ± 17.39			51.79 ± 19.45		

*PTSD* Post traumatic stress disorder. Numbers in bold indicate significant *p*-values

### Multivariable analysis

The results of a first linear regression (enter method), taking the PTSD from the Beirut blast score as the dependent variable, showed that more avoidant coping (Beta = 0.52) and more anxiety (Beta = 0.62) were significantly associated with more PTSD from the Beirut Blast (Table 4, Model 1).

**Table 4:** Multivariable analyses

**Model 1: Linear regression taking the PTSD from the Beirut blast score as the dependent variable**				
**Variable**	**Unstandardized Beta (B)**	**Standardized Beta (β)**	***p***	**95% CI**
Avoidant coping	0.52	0.21	**< 0.001**	0.24–0.80
Approach coping	0.03	0.02	0.737	−0.15-0.21
Financial wellbeing	−0.03	−0.03	0.484	−0.11-0.05
Depression (PHQ-9 score)	0.04	0.02	0.810	−0.30-0.38
Stress (BDS score)	0.23	0.11	0.152	−0.09-0.56
Anxiety (LAS score)	0.62	0.35	**< 0.001**	0.32–0.92
Personal history of anxiety disorders	−1.87	−0.05	0.304	−5.45-1.70
Personal history of PTSD	4.31	0.07	0.101	−0.84-9.46
Personal history of depression	2.21	0.06	0.223	−1.35-5.78
**Model 2: Linear regression taking the PTSD from the economic crisis score as the dependent variable**				
Avoidant coping	0.56	0.20	**< 0.001**	0.30–0.82
Approach coping	0.05	0.02	0.525	−0.11-0.22
Financial wellbeing	−0.31	− 0.29	**< 0.001**	− 0.39- -0.24
Depression (PHQ-9 score)	0.40	0.15	**0.011**	0.09–0.70
Stress (BDS score)	0.26	0.10	0.084	−0.04-0.55
Anxiety (LAS score)	0.49	0.24	**0.001**	0.21–0.77
Personal history of anxiety disorders	0.33	0.01	0.843	−2.92-3.58
Personal history of PTSD	1.90	0.03	0.425	−2.78-6.59
Personal history of depression	0.16	0.004	0.921	−3.08-3.41

Numbers in bold indicate significant p-values; Variables entered in both models: Stress (BDS score), Personal history of PTSD, Personal history of anxiety disorder, Personal history of depression, Depression (PHQ9 score), Anxiety (LAS score); *PTSD* Post traumatic stress disorder. Nagelkerke R^2^ = 41.7% (model 1) and 63.0% (model 2)

A second linear regression, taking the PTSD from the economic crisis score as the dependent variable, showed that more avoidant coping (Beta = 0.56), depression (Beta = 0.40) and anxiety (Beta = 0.49) were significantly associated with more PTSD from the economic crisis, whereas more financial wellbeing (Beta = − 0.31) was significantly associated with less PTSD from the economic crisis (Table 4, Model 2).

## Discussion

To the best of our knowledge, there have been no studies in the current scientific literature to assess the correlation between PTSD and factors such as avoidant coping, depression, anxiety, and financial wellbeing in a sample of Lebanese university students, especially in the settings of two major traumas: the economic crisis and the Beirut Blast. The multivariable analyses results showed that more avoidant coping and more anxiety were significantly associated with more PTSD from the Beirut Blast and the economic crisis, whereas more financial wellbeing was significantly associated with less PTSD from the economic crisis.

In the current study, the results showed significant rates of PTSD, respectively, from the COVID-19 pandemic (31.5%), the Beirut blast (26.0%), and the economic crisis (40.3%). Concomitantly with other studies comparing prevalence values between university students’ samples and the general population, the latter values are hardly comparable with values from the Lebanese general population. In fact, almost all the studies from Lebanon about PTSD were cross-sectional, and focused on specific sub-groups from the population [24], such as South Lebanese civilians [48], battered women [49], victims of blasts [50], and hospitalized men after armed conflicts [51]. Thus, a wide range of PTSD prevalence estimates was found, varying from 2 to 98% [24]. Another hardship would be as well, to compare the rates of PTSD that we found in our sample to other values from other university students’ samples. In fact, while 14.1% of college students who experienced a major earthquake, developed PTSD in a sample of Chinese university students [52], 2.7% of a large Chinese college student sample experienced PTSD one month after the outbreak of the COVID-19 epidemic [53], and about 9% of 3014 newly matriculated US students [27] have met criteria for PTSD. However, these wide variances are to be explained by multifaceted factors, of which the “potentially traumatic event’s load/number of events” [8, 54], and whether the traumatic event is collective or interpersonal, and many other factors, differentiating the Lebanese university student’s population from any other population.

More avoidant coping correlated with more PTSD symptoms, whether from the Beirut blast event or from the recent economic crisis. In fact, prior findings from cross-sectional studies have shed the light on the tight correlation between avoidant coping and PTSD symptoms [55], in samples of war veterans [56], motor vehicle accident survivors [57], victims of sexual and nonsexual assaults [58], and hurricane flood survivors [59]. Other few longitudinal studies [60–62] predicted a moderating relationship between avoidant coping and PTSD symptoms, whether acutely or distantly to the traumatic event. In a longitudinal study [55] targeting PTSD after exposure to domestic violence, avoidant coping was found to predict more PTSD symptoms over time, independently of the level of intimate partner violence. Indeed, the current study was conducted around one year after the Beirut Blast, and 21 months from the beginning of the economic crisis. However, given the cross-sectional design of our study, the prediction of the severity of the PTSD symptoms by the avoidant coping cannot be discussed.

More anxiety was significantly associated with more PTSD from both the Beirut Blast and the economic crisis, while more depression was significantly associated with more PTSD from the economic crisis. In fact, in a report six months after the Beirut blast, increased need for mental health consultations was seen in the Lebanese population, not just after the collective trauma of the Beirut blast, but even months before, because of the collapsed economic situation in Lebanon with a consequent unemployment rate of more than 30%, and hyperinflation of food and basic medications’ prices because of devaluation of the Lebanese pound [63]. Thus, an increased prevalence of anxiety and depression was reported as well as higher incidents of suicides and calls to suicide hotlines. Many studies have reported a significant and frequent comorbidity among anxiety, depression, and PTSD [64, 65]. In 2018, a study about the relationship between PTSD and other psychiatric comorbidities [14], 40.8% of the subjects that were diagnosed of a mixed anxiety-depression disorder have reported at least one traumatic event exposure during their lifetime.

Many findings in the scientific literature have identified common neurobiological and psychological grounds for anxiety and PTSD [66, 67]. In subjects with a higher anxiety levels, the autonomic nervous system tends to overreact in the setting of traumatic events [68]. In a recent meta-analysis [69], there was neuroimaging evidence about common brain mechanisms between anxiety disorders and PTSD with certain interrelationships explaining some emotional dysregulation symptoms that extend beyond the simple exaggerated fear response. Furthermore, one other study [70] has suggested that anxiety may be a precursor of PTSD. Hence, our current results seem to strongly align with other research findings concerning the association between anxiety and PTSD.

Similarly, another common neurobiological basis was found between PTSD and depression [71]. In a study targeting the biological differences between trauma-related depression and PTSD [72], depression and PTSD, whether comorbid or not, were both found to participate in the suppression of the hypothalamo-pituitary-adrenocortical axis in traumatized subjects. Kessler et al. [73] have found a 49% rate of depression that was frequently comorbid with PTSD. Thus, in accordance with other literature findings, it was shown in the current study, that subjects with PTSD would have higher scores of depressive symptoms.

More financial wellbeing was significantly associated with less PTSD from the economic crisis. Although the same Lebanese population may seem to be struck by the same collective stressor, the economic crisis, the response and effects of such stressor may vary in function of many factors, of which, the financial and economic differences between subgroups of this same population. In fact, low socioeconomic status was found to be one of the several risk factors associated with the development of PTSD [73, 74]. Furthermore, people with a certain financial wellbeing may seem to have a better societal safety net, which may give them a certain psychological backup/resilience to buffer the striking trauma of economic crisis [75]. In a recent study about financial well-being and post-deployment adjustment among Iraq and Afghanistan war veterans [76], it was reported that financial strains and PTSD are mutually reinforcing, since subjects with PTSD, hence with poor adaptive mechanism, will hardly ensure their financial wellbeing. Furthermore, poor financial wellbeing would deprive subjects from tools to absorb there traumas [76]. However, in some other studies, it was found that affluent countries were more likely to be affected by PTSD than poor countries, given that poor countries are of a greater “resilience reserve”, given their frequent exposure to repeated traumas [77–79]. Thus, more studies are needed to explain this correlation between PTSD and financial wellbeing, especially in the Lebanese population, where repeated traumas have granted the population a certain “bounce-back –resilience”, that may however, start to be depleted after the latest several traumatic events [77–79].

### Clinical implications

Our findings demonstrated considerable rates of PTSD among college students, whether correlated with coping strategies, anxiety, depression, or financial well-being. Thus, our data could orient health managers and universities (academic and teaching levels) to categorize specific risk factors and implement evidence-based interventions to predict the risk of the onset of PTSD. They could also provide preventive and diagnostic measures, as well as regular screenings in order to deliver subsequent high-quality care for the affected population. On another note, psychiatric caregivers and nurses, more than any other caregivers are concerned with occupational vulnerabilities, such as job burnout and compassion fatigue [80]. On the Lebanese national scale, just three mental hospitals and five units of psychiatry within general hospitals were operating prior to the Beirut blast [81]. Following the Beirut blast, and the current economic crisis, the need for mental health services has subsequently increased as well [63]. Hence, this would expose the psychiatric caregivers to further clinical exposure to mental illnesses’ emergence, such as PTSD and its correlates. Moreover, it would be a call to show more interest in possible subsequent occupational vulnerabilities that would be added to psychiatric caregivers in the settings of increased mental illnesses’ emergence.

### Limitations

There are some limitations to this study. Even though our data revealed significant associations between PTSD and many correlates, the study’s cross-sectional design prevents us from establishing causality. The data was collected online via Google Forms due to the COVID-19 pandemic and social distancing, which may have led to selection bias. For a variety of reasons, the results cannot be considered typical of the general population: women were disproportionately represented, which is expected because women are more likely to respond to surveys than men [82, 83]. This gender difference could have led to an overestimation of the rates of PTSD in our sample knowing that gender differences are well known in this mental illness with a 2:1 ratio, favoring women [84]. Plus, several sociodemographic factors were not fairly distributed, which further limits the representativeness of our sample. In addition, the percentage of participants with a personal history of depression and anxiety disorders were higher than those with a personal history of PTSD. The symptoms were subjective and not evaluated by a healthcare professional; because reporting was subjective rather than clinically based, information bias is likely. During the data collection period, there were still numerous deaths from the pandemic: more than 700 from May to August, according to official records. This might have caused the pandemic to influence our responses. Some of the scales used (PTSD, financial wellbeing, brief cope) are not validated in Lebanon, that’s why the results should be interpreted with great caution. Because of the above-mentioned limitations, our results can’t be generalized. Further research taking into account these limitations is needed.

## Conclusion

To conclude, significant correlations were found between PTSD and factors such as avoidant coping, depression, anxiety and financial wellbeing, in a sample of Lebanese university students. However, these results remain limited to a restrained sample among this age group. Nonetheless, such findings must raise the attention to serious mental and psychosocial alteration endured by Lebanese youth, that are still under fatal cumulative traumatic events, that were and even may be, intergenerationally and unintentionally transmissible [85], therefore, affecting not only the present, but also the future of a whole nation. Thus, in a country where treating mental disorders is much lesser than most other developing countries [86], this study would shed the light on the need for clinical interventions owing to detect mental vulnerabilities in the general population. Thus, longitudinal, and prospective studies to understand the multifactorial causes and correlates of PTSD, whether from previous collective traumas, or from individual traumatic history, are needed. Preventive and diagnostic procedures are to be initiated, whether acutely or distantly from the previous traumas, on a national level.

## Data Availability

All data generated or analyzed during this study are not publicly available due to restrictions from the ethics committee. The dataset supporting the conclusions is available upon request to the corresponding author (SH).
